# Kukoamine A activates Akt/GSK-3β signaling pathway to inhibit oxidative stress and relieve myocardial ischemia-reperfusion injury

**DOI:** 10.1590/acb370407

**Published:** 2022-07-22

**Authors:** Han Xu, Guibin Zhang, Long Deng

**Affiliations:** 1 PhD. Gansu Provincial Central Hospital – Department of Cardiology – Gansu Province, China.; 2 PhD. Gansu Provincial Central Hospital – Department of Integrated Pediatric Medicine – Gansu Province, China.; 3 PhD. The First Hospital of Lanzhou University – Department of Ultrasound – Gansu Province, China.

**Keywords:** Oxidative Stress, Myocardial Infarction, Reperfusion Injury, Rats

## Abstract

**Purpose:**

Myocardial ischemia/reperfusion (MI/R) injury refers to a pathological condition of treatment of myocardial infarction. Oxidative stress and inflammation are believed to be important mechanisms mediating MI/R injury. Kukoamine A (KuA), a sperm, is the main bioactive component extracted from the bark of goji berries. In this study, we wanted to investigate the possible effects of KuA on MI/R injury.

**Methods:**

In this experiment, all rats were divided into sham operation group, MI/R group, KuA 10 mg + MI/R group, KuA 20 mg + MI/R group. After 120 min of ischemia/reperfusion treatment, left ventricular systolic pressure (LVSP), left ventricular end-diastolic pressure (LVEDP), maximal rates of rising and fall of left ventricular pressure (±dp/dtmax), and ischemic area were detected. Serum samples of rats in each group were collected. The enzyme activities of catalase (CAT), glutathione peroxidase (GSH-PX), superoxide dismutase (SOD), levels of malondialdehyde (MDA), CK muscle/brain (CK-MB), tumor necrosis factor (TNF), interleukin-1β (IL-1β), and interleukin-6 (IL-6) were detected using enzyme-linked immunosorbent assay (ELISA). The apoptosis of myocardium in each group was detected according to the instructions of the terminal deoxynucleotidyl transferase dUTP nick end labeling (TUNEL) assay. The expressions of mammalian target of glycogen synthase kinase-3β (GSH-3β) and protein kinase B (Akt) mRNA level in myocardial tissues were detected via reverse transcription-polymerase chain reaction (RT-PCR).

**Results:**

MI/R rats showed a significant increase in oxidative stress and inflammation. In addition, we showed that KuA significantly improved the myocardial function such as LVSP, left ventricular ejection fraction, +dp/dt, and -dp/dt. Here, it attenuated dose-dependent histological damage in ischemia-reperfused myocardium, which is associated with the enzyme activities of SOD, GSH-PX, and levels of MDA, IL-6, TNF-α, L-1β.

**Conclusions:**

KuA inhibited gene expression of Akt/GSK-3β, inflammation, oxidative stress and improved MR/I injury. Taken together, our results allowed us to better understand the pharmacological activity of KuA against MR/I injury.

## Introduction

Myocardial infarction (MI) is defined as myocardial cell necrosis in a clinical setting, associated with acute myocardial ischemia, recurrent or persistent chest pain, severe 12-lead electrocardiogram (ECG) depression, congestive heart failure, and hemodynamic, with high mortality and disability worldwide, and also imposes a serious economic burden^1^. The main pathological symptom of coronary artery disease is a myocardial injury caused by reperfusion ischemia, leading to myocardial cell death (apoptosis, necrosis), and heart failure^2^. Reperfusion therapy is an effective treatment for selected patients with acute MI^3^. However, blood reperfusion increases mortality, tissue damage, and cardiac mechanical impairment in patients with acute myocardial infarction, which is defined as MI/reperfusion (MI/R) injury^4^.

There are several theories associated with the pathogenesis of MI/R injury: overloading of cellular Ca2^+^, formation of “white cell plugs,” impaired vascular relaxation, and a “no-reflow” phenomenon resulting from cell swelling. Oxidative stress is one of the main pathological mechanisms of reperfusion injury. It causes myocardial cell apoptosis, autophagy, inflammation, and other injuries through various pathways, resulting in irreversible myocardial cell damage and cardiac dysfunction^5^. For instance, it induces the production of reactive oxygen species (ROS) that damage cardiomyocytes^6^. Accumulating evidence suggests that mitochondrial accumulation of ROS may lead to cardiomyocyte apoptosis and necrosis^7^^,^ 8. Therefore, MI/R injury can be alleviated by preventing oxidative stress-induced cardiomyocyte damage.

Kukoamine A (KuA), a sperm kaloid, is the main bioactive component extracted from the root bark of *Lycium chinense*. KuA has several pharmacological effects, such as hypotensive, anti-inflammatory, anti-pain reliever, antibacterial, autoimmune enhancing and neuroprotective^9^. KuA plays an important role in different diseases such as fatty liver, diabetes, and radiation-induced brain injury^10^^,^ 11. Among them, KuA has been shown to have strong antioxidant activity, which can significantly attenuate hydrogen peroxide (H_2_O_2_)-induced apoptosis in TAR DNA-binding protein 43 (TDP-43)-induced neuroblastoma (SH-SY5Y) cells by inhibiting oxidative stress and inactivating apoptosis signaling pathway^12^. KuA also increased the activities of brucella Cu-Zn superoxide dismutase (Cu-Zn SOD) and manganese superoxide dismutase (Mn-SOD) and decreased malondialdehyde (MDA) and H_2_O_2_. After mild cerebral ischemia treated with KuA, the expression of caspase-3 and cytochrome was increased, and the ratio of Bcl-2 associated X protein/B cell lymphoma 2 (Bax/Bcl-2) was significantly decreased^13^. However, whether KuA plays a protective role against MI/R injury is unknow.

Activation of the protein kinase B/glycogen synthase kinase (Akt/GSK-3β) pathway could regulate the development of many diseases. For example, it regulates natural killer (NK) cell activity and cancer cell sensitivity to NK cells, leading to increased breast cancer and lung metastasis^14^. Mitochondrial coenzyme Q protects rats from sepsis-induced acute lung injury by activating the phosphatidylinositol 3-kinases/activation of the protein kinase B/glycogensynthase kinase -3beta/mammalian/mechanistic target of rapamycin (PI3K/Akt/GSK-3β/mTOR) pathway^15^. Phosphoinositide 3-kinase/protein kinase B/nuclear factor-kappa β (PI3K/Akt/NF-β) signaling pathway plays a role in cardiovascular and lung injury and may mediate apoptosis in lung cells. GSK-3β, a signal transduction factor, is also involved in cardiopulmonary-induced apoptosis in lung cells^16^. This controversy is neuroprotective against depletion and neurotoxicity of primary cortical neurons due to neurodegenerative diseases in N-methyl-D-aspartate (NMDA) receptor subunit 2B (GluN2B), containing protein cultures and phosphorylated receptors that bind to the PI3K/Akt/GSK-3β pathway^17^.

In this study, the MI/R injury model was first established. Moreover, we investigated the effects of KuA on MI/R, as well as its relationship with Akt/GSK-3β signaling pathway, particularly its effect on reducing oxidative stress and inflammatory reaction.

## Methods

This study was approved by the animal care review committee of Gansu Provincial Central Hospital and the institutional review board of the University of Connecticut carried in Gansu Provincial Central Hospital and followed by National Academies Press Guide for the Care and Use of Laboratory Animals.

Female Wistar rats (250 to 280 g) were obtained from Liaoning Changsheng Biotechnology Company. Moreover, four or five rats were kept in each cage with free access to food and water and exposed to a 12/12 h light-dark cycle at 21 to 23°C with a relative humidity of 55 to 65% in clear plastic cages (55 × 40 × 20 cm) for one week before the initiation of the experiment. The management of this study was approved by the Ethics Committee of Gansu Provincial Central Hospital (GSCH 20190202).

### Reagents

KuA was obtained from Chengdu Biopurify Phytochemicals Ltd. (China) with a purity of more than 98.22%. KuA was dissolved in dimethyl sulfoxide (DMSO) (50 mM) for storage and further dissolved using a culture medium before administration to cells with the final concentration of DMSO < 0.1% (v/v). Dulbecco’s Modified Eagle’s Medium (DMEM), Neurobasal medium, B27, glutamine, horse serum, and wortmannin were obtained from Gibco Life Technologies (Grand Island, NY, United States of America).

### Experimental protocol

After sham treatment or MI/R treatment, rats received either KuA dissolved in saline or the same volume of saline alone (vehicle, 0.5 mL/kg) via intravenous injection (i.v.) immediately. KuA was administered at doses of 5, 10, and 20 mg/kg body weight, respectively. Based on the dose of KuA administration, rats were randomly divided into four groups: sham group with vehicle treatment (n=10), MI/R with vehicle treatment (n=10), MI/R with KuA treatment of 10 mg/kg i.v. (n=10), and MI/R with KuA treatment of 20 mg/kg i.v. (n=10). The KuA doses applied lasted for four weeks in this work. The doses of KuA were selected based on previous studies^10^.

### Establishment of myocardial ischemia-reperfusion injury model

Wistar rats were anesthetized with 3% pentobarbital sodium (50 mg/kg). The heart was exteriorized through an open chest surgery. A 6/0 nylon suture was inserted to make a slipknot around the left anterior descending artery (LAD) and induce myocardial ischemia. The slipknot was released after 30 min of ischemia, and myocardial cells were reperfused for 6 h (to analyze cardiomyocytes apoptosis and measure the myocardial infarction size) and 72 h (to determine cardiac function). The rats in the sham group underwent the same operation, but without occluding the suture under LAD. After reperfusion, the rats were sacrificed by intraperitoneal injection of sodium pentobarbital (200 mg/kg). Blood samples and cardiac tissues were harvested for further study.

### Echocardiography and hemodynamic detection

Two-dimensional and M-mode echocardiographic measurements were performed using a Vevo 770 high-resolution echocardiographic system (Visual Sonics Inc., Toronto, Canada) in standard settings, four weeks after the MI/R operation. The parameters that were assessed were as follows: left ventricular (LV) ejection fraction. A plastic catheter was first inserted into the left ventricle along the common carotid artery. Subsequently, left ventricular systolic pressure (LVSP), left ventricular end-diastolic pressure (LVEDP), and maximal rates of rising and fall of left ventricular pressure (dp/dtmax) were recorded using a physiological recorder.

### Determination of myocardial infarct size and apoptosis

The central area of myocardial infarction was first found. Then, the apex of the heart parallel to the atrioventricular groove was cut, followed by incubation with triphenyl tetrazolium chloride (TTC) solution (Oxoid, Hampshire, United Kingdom) for 15 min. Subsequently, the infarction area appeared white and could be distinguished from normal myocardial tissues. Ischemic and non-ischemic areas were separated, and the volume ratio of the infarction area to the left ventricle was calculated as the myocardial infarction area. For measuring the deoxyribonucleic acid (DNA) nicks, the TdT-mediated dUTP-biotin nick end labeling (TUNEL) assay was used to reflect the level of intracellular apoptosis. Cardiac tissues were fixed in 10% neutral buffered formalin solution and embedded in paraffin wax, after which they were cut into 5-μm sections. The number of apoptotic cardiomyocytes was determined using TUNEL immunofluorescence staining, which was performed with the paraffin sections. Apoptosis index was calculated using the Eq. 1:


Apoptotic cardiomyocytes = [Number of TUNEL-Positive cells]/Total cells x 100% (1)

### Determination of activities of myocardial enzyme in serum

The blood samples were collected 6 h after reperfusion, clotted at 37°C for 1 h, and then centrifuged (1,200 × g) for 15 min. The supernatants of each group were removed and stored at -80°C for further analysis. The activities of creatine kinase isoenzyme (CK-MB) were determined using diagnostic kits with a biochemistry analyzer as per the manufacturer’s protocols.

### Measurement of superoxide production and catalase, glutathione peroxidase, superoxide dismutase, and malondialdehyde in tissues

Myocardial superoxide production was assessed by lucigenin enhanced chemiluminescence^18^. A cardiac tissue piece was weighed accurately. Phosphate buffered saline containing protease and phosphatase inhibitors were added (w/v = 1:9). After homogenization, the mixture was centrifuged at 180 × g at 4°C for 5 min. The collected supernatant was added with nicotinamide adenine dinucleotide phosphate (NADPH; 1 mmol/L) and luster concentrate (50 μmol/L). The activity of ligninase was measured using a chemiluminescence analyzer. The production of myocardial superoxides was expressed as relative light units per second per milligram of the heart. The myocardial tissue was excised and homogenized in 0.9% sodium chloride (w/v = 1:9) and then centrifuged (2,200 × g) for 10 min at 4°C, and the supernatants were taken to determine myocardial catalase (CAT), glutathione peroxidase (GSH-Px) and superoxide dismutase (SOD) activities, and MDA content using spectrophotometric assay kits following the manufacturer’s protocols.

### Measurement of interleukin-1beta, interleukin-6, and tumour necrosis factor-alpha in tissues

Rats have sacrificed 6 h after the reperfusion. The activities and messenger RNA (mRNA) levels of interleukin-1beta (IL-1β), interleukin-6 (IL-6), and tumour necrosis factor-alpha (TNF-α) were measured by rapidly dissecting the heart tissue. The activities of IL-1β, IL-6, and TNF-α from the myocardial tissue homogenate were measured using enzyme-linked immunosorbent assay (ELISA) kits by following the manufacturer’s protocols. Total RNA was extracted from cardiac tissues using TRIzol (Thermo Fisher, Waltham, MA, United States of America) following the manufacturer’s instructions. The total RNA was quantified using a T-6 Series UV–Vis spectrophotometer, and RNA was reverse transcribed into complementary DNA by using the Hifair II First Strand cDNA Synthesis kit (Yeasen Biotech, China). RNA was then reverse-transcribed with oligo (dT) primers, and a quantitative real-time polymerase chain reaction (qPCR) was performed using gene-specific primers using an SYBR Green Master Mix kit (Yeasen Biotech, China). The primers were as follows: IL-1β forward primer 5-GACTTCACCATGGAACCCGT-3 and reverse primer 5-CAGGGAGGGAAACACACGTT-3; IL-6 forward primer 5-GACTTCCAGCCAGTTGCCTT-3 and reverse primer 5-GCAGTGGCTGTCAACAACAT-3; TNF-α forward primer 5-CTTCTCATTCCCGCTCGTGG-3 and reverse primer 5-CTTGTTGGGACCGATCACCC-3; and β-actin forward primer 5-GCACCGCAAATGCTTCTAGG-3 and reverse primer 5-AAAGGGTGTAAAACGCAGCTC-3. Data were calculated using the 2^-↑CT^ method and normalized to the expression of β-actin.

### Statistical analysis

The results were presented as means ± standard deviation. Two-way analysis of variance, followed by Bonferroni’s post hoc test, and individual t-tests were analyzed using the GraphPad Prism software version 5.04 (GraphPad Software, San Diego, CA, United States of America). A p-value of <0.05 was considered significant.

## Results

### Effect of KukA on cardiac hemodynamic changes and infarction area after ischemia-reperfusion treatment in rats

**Figure 1:**
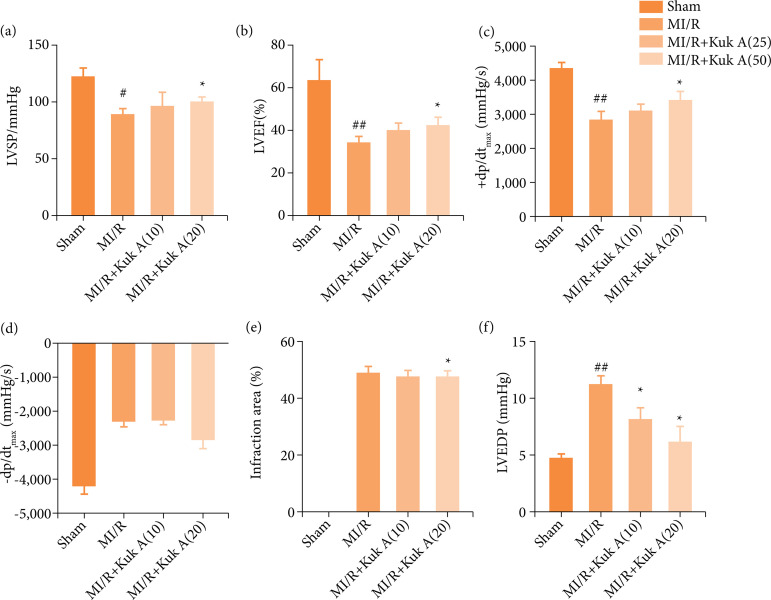
Effects of KukA on cardiac hemodynamic changes and infarction area after MI/R treatment in rats.

KukA: Kukoamine A; MI/R: myocardial ischemia/reperfusion; LVSP: left ventricular systolic pressure; LVEF: left ventricular ejection fraction; dp/dt: rates of rising and fall of left ventricular pressure; LVEDP: left ventricular end-diastolic pressure.

As shown in Fig. 1, LVSP, left ventricular ejection fraction (LVEF), +dp/dt, and -dp/dt were decreased significantly in MI/R when compared with those of the Sham group. However, LVEDP in the MI/R group was remarkably higher than that of the Sham group (p<0.01). After the administration of KukA 10 mg for the last four weeks, LVEDP decreased significantly more than MI/R group, whereas LVSP, LVEF, +dp/dt, -dp/dt and infarction area were not significantly different than MI/R group. After the treatment with KukA 20 mg for last four weeks, LVSP, LVEF, +dp/dt, and -dp/dt were decreased than the MI/R group, while infarction area and LVEDP were significantly decreased than MI/R group. This suggested that KukA could improve cardiac function and prevent MI on the mentioned indexes.

### Effect of KukA on oxidative stress and myocardial cell apoptosis after ischemia/reperfusion treatment in rats

**Figure 2:**
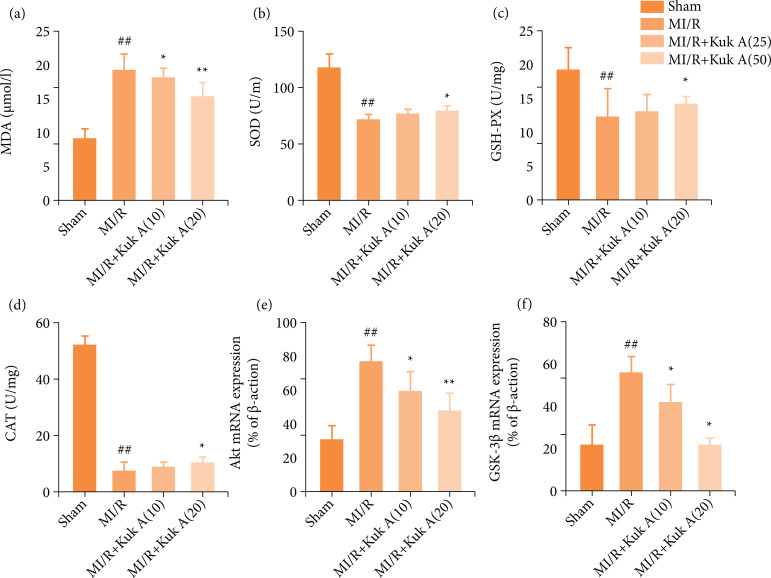
Effect of KukA on oxidative stress and Akt/GSK-3β after MI/R treatment in rats.

MDA: malondialdehyde; SOD: superoxide dismutase; MI/R: myocardial ischemia/reperfusion; KukA: Kukoamine A; GSH-PX: glutathione peroxidase; CAT: enzyme activities of catalase; Akt: protein kinase B; GSK-3β: glycogen synthase kinase-3β.

It was found that, compared with the Sham group, MDA and superoxide generation level in the MI/R group were markedly increased, whereas the enzyme activities of SOD, CAT, and GSH-PX were significantly decreased than the MI/R group. Compare with the MI/R group, MDA level was decreased markedly in MI/R+KukA 10 mg group, whereas no significant difference was seen among enzyme activities of SOD, GSH-PX, and CAT in the MI/R group and MI/R+KukA 10 mg group. After the treatment with KukA 20 mg for the last four weeks, MDA and superoxide generation levels were decreased significantly than the MI/R group, but enzyme activities of SOD, GSH-PX, and CAT were increased markedly than the MI/R group (Fig. 2). The results manifested that no TUNEL-positive cells were found in the Sham group. Moreover, the percentage of apoptosis cells also decreased significantly in MI/R+KukA 20mg than MI/R group (Fig. 2c).

### Effect of KukA on Akt/GSK-3β pathway after I/R treatment in rats

As shown in Fig. 2, the levels of Akt and GSK-3β in the MI/R group were evidently higher than those of the Sham group (p<0.01). However, the level of these indexes was more significant than those of the MI/R group after KukA 10 and 20 mg administration based on the MI/R model. In this study, the results revealed that, after the establishment of the MI/R model, KukA treatment could decrease markedly the levels of Akt and GSK-3β mRNA.

### Effect of Kukoamine A on inflammation factors and creatine kinase isoenzyme after ischemia/reperfusion treatment in rats

**Figure 3:**
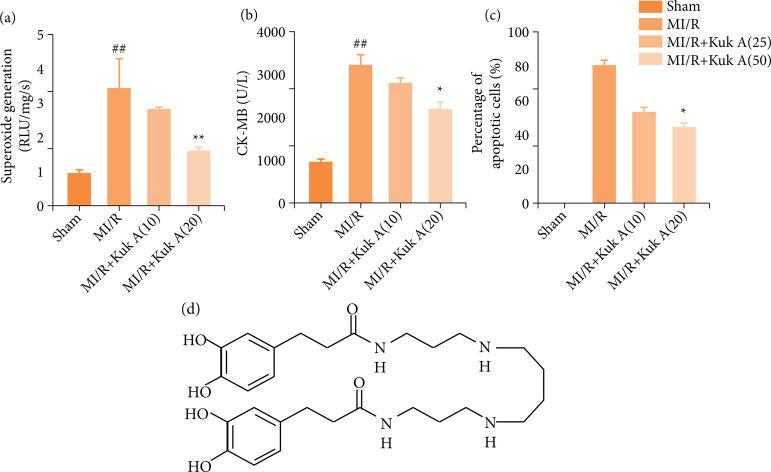
Effect of KukA on superoxide generation, CK-MB, and percentage of apoptotic cells after I/R treatment in rats.

**Figure 4:**
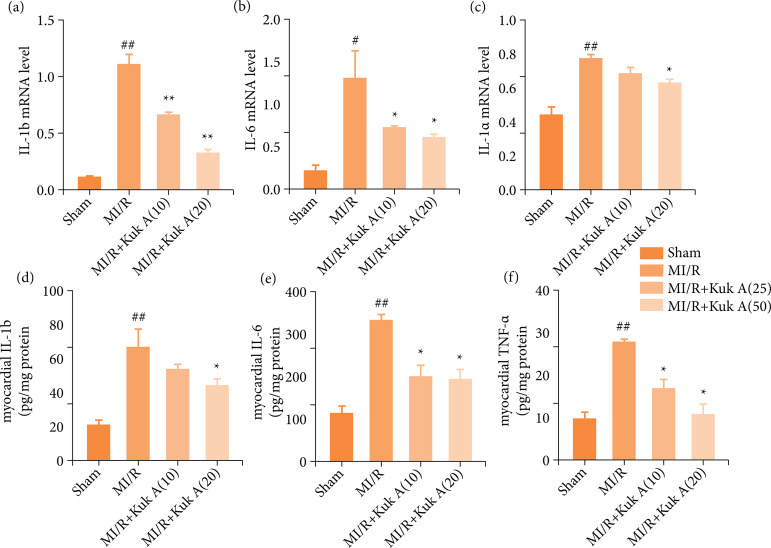
Effect of KukA on inflammation factors after MI/R treatment in rats.

As shown in Fig. 3, the mRNA and protein levels of IL-1β, CK-MB, TNF-α, and IL-6 in the MI/R group were significantly higher than those of the Sham group. However, the IL-1β and IL-6mRNA levels and TNF-α and IL-6 protein levels were decreased markedly after KukA 10 mg administration in MI/R rats. After the combination of KukA 20 mg with the MI/R group, the mRNA and protein levels of IL-1β, CK-MB, TNF-α, and IL-6 in the MI/R group were significantly lower than MI/R group. The mentioned results suggested the effects of KukA on the expression levels of CK-MB, TNF-α, and IL-6 (Fig. 4).

MI/R: myocardial ischemia/reperfusion; KukA: Kukoamine A; CK-MB: creatine kinase isoenzyme; I/R: ischemia/reperfusion.

KukA: Kukoamine A; MI/R: myocardial ischemia/reperfusion; IL-1β: interleukin-1β; IL-6β: interleukin-6β; TNF-α: tumor necrosis factor α.

## Discussion

At present, the treatment of coronary heart disease is mainly to improve coronary artery perfusion through surgery or medicine. However, ischemic myocardial cells usually release a large number of inflammatory mediators during the process of blood flow reperfusion. This may cause metabolic dysfunction of myocardial cells, degeneration and necrosis of myocardial cells and oxidative stress reaction. MI/R injury has a wide range of side effects that partially offset the benefits of early revascularization in acute MI^19^. Therefore, an in-depth study on the mechanism and treatment of MI/R is of far-reaching significance for improving the prognosis of cardiovascular diseases^20^^,^ 21.

Application of ischemic postconditioning in MI is temporary occlusion of blood flow to the infarcted myocardium in predetermined sequential phases by balloon inflation and echocardiography after the successful reopening of the causative artery^22^^,^ 23. Quantitative assessment of myocardial damage and the outcome of any intervention to reduce myocardial damage can directly measure myocardial damage in dead animals, or indirectly in humans through imaging or biomarkers. Imaging is best performed with magnetic resonance imaging (MRI), and biomarkers can be used as surrogate scores for heart rate assessment. Troponin is currently the gold standard for diagnosing myocardial infarction, but creatine kinase (CK) and creatine kinase-MB (CK-MB) are used as important biomarkers to characterize myocardial injury in I/R injury studies^24^^,^ 25.

Oxidative stress and reductive stress are two dynamic stages that cells undergo when adapting to harmful endogenous or exogenous stimuli. The production and release of oxidative and reductive stress also play important role in MI/R injury. Numerous preclinical and clinical studies have demonstrated the cardioprotective effects of antioxidant therapy, thereby highlighting the role of ROS-mediated stress injury in ischemic myocardial injury/reperfusion pathology^26^^-^ 28. The level of MDA and enzyme activities of SOD, GSH-PX, and CAT were also the markedly oxidative and reductive stress biomarker based on previous studies^29^. Several research projects aim to reduce the level of ROS by activating the antioxidant defense system to neutralize the effects of reactive oxygen species on the structure of living cells, especially the role of SOD as a free radical scavenger in animal experiments^30^. Therefore, the first research option is an enzyme capable of neutralizing ROS, the main research direction are SOD and glutathione peroxidase type 1 (GPx)^31^.

Growing evidence implicates inflammation in the pathophysiology of MI/R injury^32^. IL-1β is an important early inflammatory mediator in I/R injury (IL-1β)^33^. I/R induces IL-1β expression in the heart, and the inhibition of IL-1β prevents myocardial injury after I/R, suggesting that the deleterious effects of myocardial I/R are mediated, at least in part, by IL-1β^34^. Jong et al. investigated that IL-6 contributes to early infarction after reperfusion due to infarction, local and systemic inflammation, neutrophil infiltration, coagulation, and ST-elevation myocardial infarction^35^. TNF-α, a major anti-inflammatory factor, is excessively released during MI/R, promoting cardiac insufficiency and apoptosis^36^. Most studies using pharmacological strategies to inhibit TNF-α showed improved myocardial function and reduced infarct size after MI/R injury. Drugs capable of neutralizing TNF-α, such as etanercept, a fusion protein of soluble TNF-α receptor 2 and IgG1 FC-terminal, have been used clinically to treat diseases such as rheumatoid arthritis and psoriasis^37^. This finding broadly supports the work of other studies in this area linking the level of inflammation factors and MI/R.

KuA (Fig. 4d), a main bioactive ingredient in Cortex Lycii Radicis, has been shown to have antihypertensive effects in vivo^38^. Previous studies have shown that KuA reduces oxidative stress and excitotoxicity and may improve radiation-induced brain damage^12^^,^ 39. These studies show that KuA has strong biological activity, especially in neuroprotection and fatty liver^10^^,^ 11 ^,^ 17. However, whether KuA showed the activity of anti-MI/R injury is still unknown. Therefore, in our study, we found that KukA plays protect role in MI/R injury. Besides, we also found KukA can against oxidative stress and inhibit inflammation in this process, which was in accordance with previous studies^12^^,^ 39.

## Conclusion

KuA inhibits Akt/GSK-3β gene expression and suppresses inflammation and oxidative stress. Taken together, our results allowed us to better understand the pharmacological activity of KuA against MI/R injury.
