# ERRATA: Kukoamine A activates Akt/GSK-3β signaling pathway to inhibit oxidative stress and relieve myocardial ischemia-reperfusion injury

**DOI:** 10.1590/acb370407.errata

**Published:** 2022-09-09

In the manuscript Kukoamine A activates Akt/GSK-3β signaling pathway to inhibit oxidative stress and relieve myocardial ischemia-reperfusion injury, which DOI is: https://doi.org/10.1590/acb370407, published in the journal **Acta Cirúrgica Brasileira**, 2022;37(04)e370407, page 1:

**Instead of**:

1. PhD.

2. PhD.

3. PhD.

**Should be**:

1. MD.

2. MD.

3. MD.

